# Machine learning predicts treatment sensitivity in multiple myeloma based on molecular and clinical information coupled with drug response

**DOI:** 10.1371/journal.pone.0254596

**Published:** 2021-07-28

**Authors:** Lucas Venezian Povoa, Carlos Henrique Costa Ribeiro, Israel Tojal da Silva

**Affiliations:** 1 Aeronautics Institute of Technology (ITA), Bioengineering Lab, São José dos Campos, Brazil; 2 Aeronautics Institute of Technology (ITA), Computer Science Division, São José dos Campos, Brazil; 3 AC Camargo Cancer Center (ACCCC), International Research and Educational Center, São Paulo, Brazil; 4 Federal Institute for Education, Science, and Technology of São Paulo (IFPS), Jacarei, Brazil; Taipei Medical University, TAIWAN

## Abstract

Providing treatment sensitivity stratification at the time of cancer diagnosis allows better allocation of patients to alternative treatment options. Despite many clinical and biological risk markers having been associated with variable survival in cancer, assessing the interplay of these markers through Machine Learning (ML) algorithms still remains to be fully explored. Here, we present a Multi Learning Training approach (MuLT) combining supervised, unsupervised and self-supervised learning algorithms, to examine the predictive value of heterogeneous treatment outcomes for Multiple Myeloma (MM). We show that gene expression values improve the treatment sensitivity prediction and recapitulates genetic abnormalities detected by Fluorescence *in situ* hybridization (FISH) testing. MuLT performance was assessed by cross-validation experiments, in which it predicted treatment sensitivity with 68.70% of AUC. Finally, simulations showed numerical evidences that in average 17.07% of patients could get better response to a different treatment at the first line.

## Introduction

Multiple Myeloma (MM) is a cancer of plasma cells, the second most common neoplasm. It is considered an incurable disease, with MM patients having a mean survival of five years [1], characterized by heterogeneity in the clinical outcome, driven by chromosomal abnormalities [2]. Although the detection of these chromosomal events allows better understanding of the genetic instability spectrum associated with the clinical outcome, the specific prognostic value of most chromosomal abnormalities is still controversial and remains challenging for the different biological subgroups [3]. Thus, the prediction of Treatment Sensitivity (TS) has long been pursued in order to make treatment choices and thus, to better allocate MM patients to alternative treatment options [4, 5].

Given the massive increase in data available to the medical research community, there are opportunities in the field of Machine Learning (ML) for new approaches regarding prognostic assessment. Hence, analysis of cancer high-throughput “omics” data in combination with clinical ones may lead to a more precise characterization of the disease and is likely to have higher clinical utility. Of note, TS prediction is one of the critical tasks and has the potential to benefit a subset of the patients who may be associated with serious side effects. However, proportional to the biological complexity associated to cancer, there are several computational challenges that should be addressed while creating models related to MM. High dimensional data with few samples require specific care about the modelling processes in order to guarantee that final patient classifiers are not overfitted [6]. The unbalanced number of samples related to each event (e.g., treatment sensitivity) is another important characteristic that increases complexity. Indeed, MM patients with good response to a treatment or long survival correspond to the rarest events. Classifying these events incorrectly is costly and can generate inapplicable models [7]. Finally, data sets are composed of several markers and are thus represented by high dimensional feature vectors, a characteristic that brings the challenge of selecting only significant and non-redundant markers in order to avoid noisy or too complex models [6].

Current approaches focus on generating combined clinical and molecular markers [8]. Despite the efforts, caution must be exercised whenever applying the markers signatures derived from specific molecular features to predict cancer outcome [9]. Nonetheless, approaches that focus on generating clinical and molecular markers that map signatures to TS may allow to customize the therapeutic regimen to each individual patient [10, 11]. In MM, a seminal study [12] proposed an algorithm called Simulated Treatment Learning (STL) and gathered data from three phase III trials to predict treatment benefit. The approach took into account a reasonable assumption that molecular data of patients who received different treatments, but who had genetically identical tumors, could be used to predict how a particular patient would benefit from an alternative treatment.

Despite the significant advances promoted by STL, an approach considering the interplay of clinical markers, gene expression levels, and treatments into the same model still remains to be evaluated. Herein, we proposed the Multi Learning Training approach (MuLT) which aggregates clinical and molecular data to predict TS. This allow us to perform simulations to estimate which treatment could maximize sensitivity response of each patient.

We applied our approach on molecular and clinical data measured from 1,525 patients with newly diagnosed MM from the Multiple Myeloma Research Foundation (MMRF) CoMMpass study [37]. We first reveal that gene expression profiling recapitulates known molecular damage detected by Fluorescence *in situ* hybridization (FISH) analysis. We further show that TS prediction accuracy increases by incorporating the gene expression levels. Next, we proposed the MuLT that combines supervised, unsupervised, and self-supervised methods to accurately predict TS. Finally, our simulations pointed out alternative first line treatment options for 17.07% of patients.

## Results

### Gene expression levels predict FISH markers with high accuracy

Our study started by assessing if the main markers employed in the current MM clinical decision-making process [13] could be predicted by gene expression levels. Thus, we employed a Simplified ML Approach (SMLA) (see Methods section for details) to create specific predictors for each FISH marker available in MMRF data set. All of these steps were performed over 10-fold cross-validation (CV) [14].

**Fig 1:**
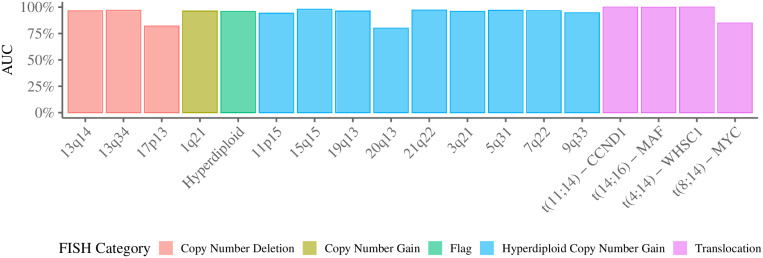
Average AUC of FISH predictors in 10-fold CV experiments. Hyperdiploid flag indicates if patients have at least two gains in the hyperdiploid gain regions [13].

Fig 1 presents the average AUC [15] associated with each FISH marker (x-axis label) predictor. Results show an average AUC of 94.53%(±5.92%) among all FISH predictors. The predictor for the *20q13* marker had the worst performance with average AUC of 80.04%(±10.11%), while the predictor for the *t(4;14)—WHSC1* marker reached 100.00%(±0.00) on the same metric. Genes used as predictors for FISH markers and detailed metrics, including train and test AUC and loss, are listed at S1 File. We conclude that gene expression levels accurately mirror FISH markers in MM patients.

### Treatment sensitivity outcome stratifies overall survival

**Fig 2:**
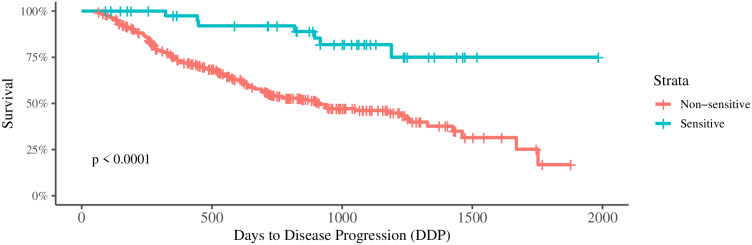
Observed survival grouped by TS classes according to days to disease progression with death events.

According to the Treatment Response (TR), patients were categorized into one of six classes: Progressive Disease (PD), Stable Disease (SD), Partial Response (PR), Very Good Partial Response (VGPR), Stringent Complete Response (SCR), and Complete Response (CR). TS is defined as a binary marker derived from TR, categorizing patients into either treatment sensitive or treatment non-sensitive classes. In our study, patients identified as SCR or CR compose the treatment sensitive class, and those in any of the remaining categories are in the non-sensitive one. The MMRF data set contains more than 700 patients data annotated with the TR outcome, and a relatively low prevalence (30%) of patients with the Days to Disease Progression (DDP) information. The reduced number of patients annotated with DDP in the MMRF cohort hampers the design of precise risk models. We overcome this by using TS as a surrogate for DDP. To further assess our approach, we apply it to stratify the DDP patients as shown in Fig 2, which shows the maximum DDP stratification capability of a perfect model used to predict TS taking the MMRF data set into account. We concluded that the proposed TS definition based on TR classes was able to stratify overall survival groups (p-value < 0.0001).

### Gene expression levels improve treatment sensitivity prediction

We used the SMLA (see Methods section for details) and performed two independent experiments in order to evaluate if gene expression levels can add information to clinical markers while predicting TS.

First, we created TS predictors taking a single clinical marker (e.g., age, race, stage) as input. Then we performed a second independent experiment, creating another TS predictor combining that single clinical marker to selected genes. All experiments were performed over 10-fold CV.

**Fig 3:**
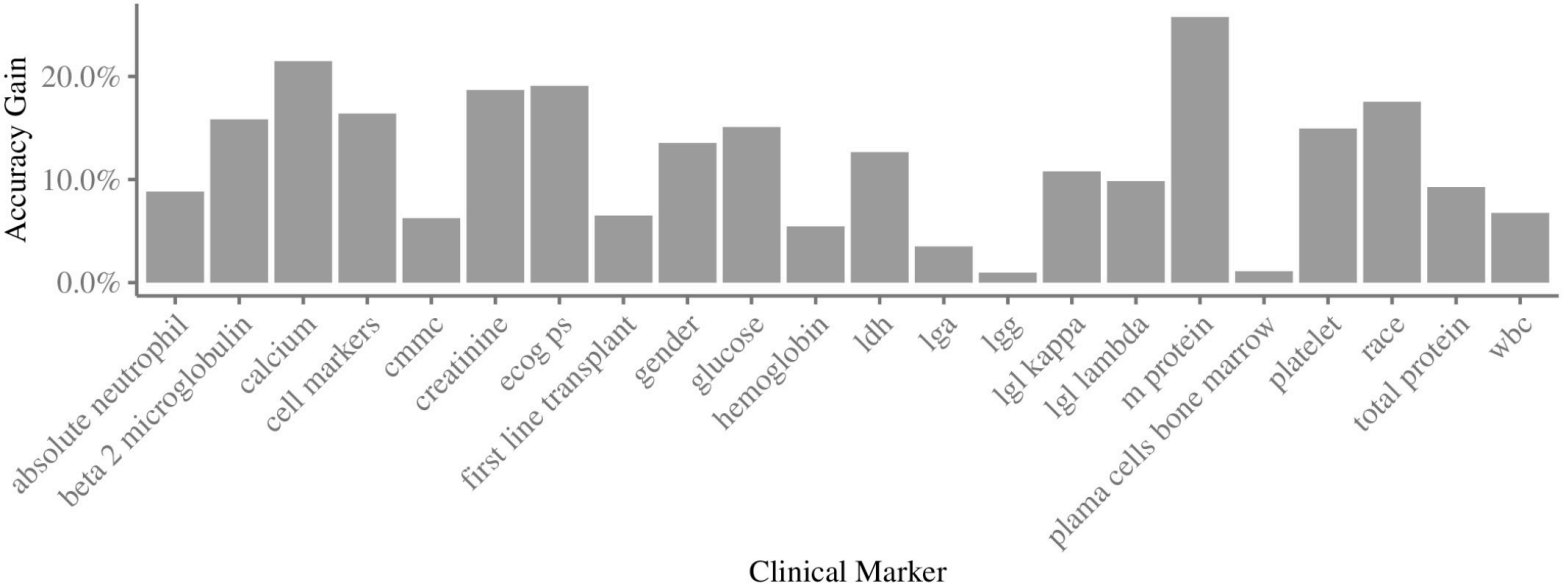
Improvements of TS prediction accuracy reached by gene expression levels combined to clinical markers. These results were obtained from two independent 10-fold CV experiments. First experiment uses a single clinical marker to estimate TS, and the second experiment combines that clinical marker with a set of selected genes.

For each clinical marker, Fig 3 shows the accuracy gain comparing two TS predictors. The first predictor consists of a single clinical marker and a set of selected genes. The second one is composed only by the same clinical marker. We observed an average gain of 11.82% (±6.65%) in accuracy. The *m protein* marker produced the largest gain of 25.76%, while the *lgg* marker had the smallest gain of 0.96%. The S2 File informs the complete list of selected genes used in these experiments. The selection itself was performed using Algorithm 1, the same feature selection algorithm of MuLT.

### Proposition of a novel machine learning approach

**Fig 4:**
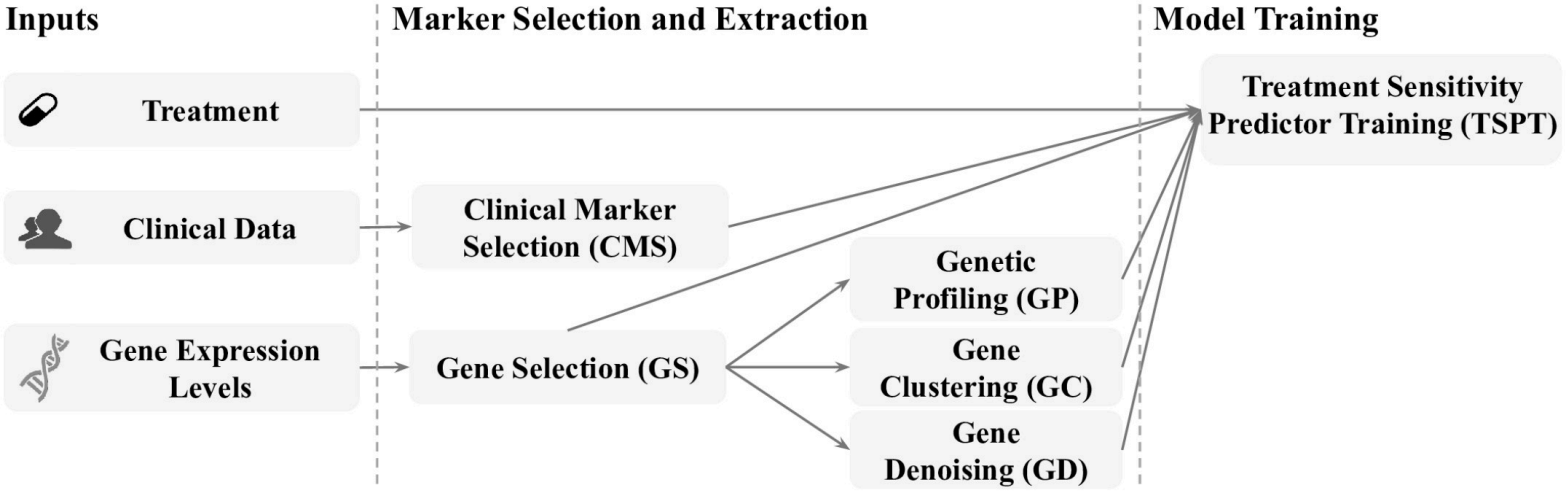
MuLT overview. It inputs clinical markers, gene expression levels, and treatments, performs a set of marker selection and extraction steps, and then creates a TS predictor.

In an attempt to improve TS prediction accuracy, we proposed MuLT, that comprises the following integrative approaches: i) combining clinical makers, gene expression levels, and treatments to compose a more sophisticated patient description; and ii) creating new representation [16] of gene expression levels based on unsupervised and self-supervised learning algorithms in order to find hidden predictive information (Fig 4). Briefly, it takes clinical data (e.g., age, race, stage, first line transplant), gene expression levels, and five first line treatments (i.e., Bor-Cyc-Dex, Bor-Dex, Bor-Len-Dex, Len-Dex, Non-treatment) as input. Then, it starts by executing the steps Clinical Marker Selection (CMS) and Gene Selection (GS), which respectively select clinical markers and genes in order to reduce noise and complexity of TS predictors. For these steps, we created an algorithm that selects predictive markers for TS, removing those with information embedded by more significant markers (see Methods section for more details). Results from GS are independently loaded into Genetic Profiling (GP), Gene Clustering (GC), and Gene Denoising (GD) steps. These aim at creating gene expressed-based features to improve the TS predictor accuracy (see Methods section for more details).

GP and GC steps were motivated by the fact that different genetic profiling and gene clusters could be associated with some transcription factors and known recurrent translocations that imply in better or worse overall survival [17]. In addition, these genetic profiles and clusters could underlie the transitions between the disease phases of MM [18]. The GD step was designed to create a noise-resilient representation of gene expression levels, which are based on a sampling process that naturally generates a noise measure.

The Treatment Sensitivity Predictor Training (TSPT) step creates a TS predictor using a supervised learning algorithm. It inputs all features previously selected and created during Marker Selection and Extraction (Fig 4). As a learning algorithm is regulated by several hyperparameters, the TSPT step applies a hyperparameter optimization using half of the training data set and the Bayesian Optimization (BO) algorithm [19] before TS predictor training takes place. After that optimization, the entire training data set is used to train the TS predictor.

The final classifier is based on the Light Gradient Boosting Machine (LightGBM) [21] algorithm and returns a value in [0, 1], namely the TS score. We estimated the optimal class threshold based on the training data set, where “optimal” refers to the threshold associated with the highest AUC computed over the training data set. A TS score greater than or equal to that threshold represents the TS sensitive class, while a TS score smaller than that threshold represents the TS non-sensitive one.

### MuLT predicts treatment sensitivity

MuLT was evaluated via 10-fold CV. We split the data set into ten folds of similar sizes. To avoid bias, we randomly equalized the number of sensitive and non-sensitive patients and treatments per fold. For each experiment, we employed nine folds to compose the training data set used to apply MuLT, and one fold to compose the validation data set used to evaluate our proposed approach. MuLT performed with an average AUC of 68.70%, ranging from 59.49% to 74.70%, with standard deviation of 4.66%.

We face the challenge of fairly comparing results to related work taking into account data set composition, available markers, and clinical outcome definition (e.g., treatment sensitivity, risk group, survival). To deal with this limitation, we carried out new experiments using SMLA with four different ML models, namely MLP [20], LightGBM [21], and Support Vector Machines (SVM) [22]. Table 1 presents the average performance on 10-fold CV experiments of the TS predictors created by MuLT and MLP, SVM, and LightGBM models embedded in SMLA. SMLA results do not reach AUC greater than 61.54%, where LightGBM achieved the best performance and SVM had the worst one with AUC of 55.57%. Taking AUC into account, MuLT results are statistically better than SMLA ones with p- value = 2.529 × 10^−5^ computed via t-test.

**Table 1:** Performance of TS predictors created by MuLT and SMLA on 10-fold CV experiments. Namely, sensitivity is the rate of correct prediction of patients identified as sensitive to first line treatments, and specificity describes the correct prediction percentage of patients identified as non-sensitive to first line treatment.

ML Model	AUC	Accuracy	Sensitivity	Specificity
MuLT	68.67%(±4.66%)	64.61%	61.70%	65.56%
SMLA + LightGBM	60.15%(±5.57%)	61.82%	51.46%	65.00%
SMLA + MLP	61.54%(±4.96%)	60.89%	56.49%	62.20%
SMLA + SVM	55.57%(±8.24%)	51.52%	57.65%	49.21%

The overall classification performance per treatment is shown in Table 2. The Bor-Len-Dex treatment reached the best AUC with 67.13%, while Bor-Cyc-Dex the worst one with 63.09%. Len-Dex presents the worst sensitivity with 28.57%, followed by Bor-Cyc-Dex with 55.17%. Specificity performance ranged from 58.48% to 86.05%, corresponding to Bor-Len-Dex and Len-Dex, respectively. See S2 File for a complete list of selected clinical markers and genes used to create TS predictors in our experiments and details about the performance metrics. Finally, it is important to highlight that both MuLT and SMLA (including its variants based on LightGBM, MLP, and SVM) were evaluated under the same raw data and CV fold arrangements.

**Table 2:** Classification performance metrics per treatment on 10-fold CV. Sample size describes the amount of patients annotated with the correspondent treatment. All CV validation data sets were combined into a unique data set to compute these metrics.

Treatment	Sample Size	AUC	Accuracy	Sensitivity	Specificity
Bor-Cyc-Dex	133	63.09%	63.16%	55.17%	65.39%
Bor-Dex	64	65.41%	76.56%	57.14%	78.95%
Bor-Len-Dex	236	67.13%	60.16%	64.61%	58.48%
Len-Dex	50	66.61%	78.00%	28.57%	86.05%
Non-treatment	232	65.93%	63.79%	55.55%	66.86%

### New marker set capable of predicting treatment sensitivity

The MMRF data set contains a total of 55,103 genes and 26 clinical markers. The marker selection is based on Algorithm 1 (see the Methods section for details), and our experiments selected 74 genes and 11 clinical markers.

Yet, a unique gene (i.e., GIHCG) and 5 clinical markers (i.e., hemoglobin, bun, beta 2 microglobulin, ldh, first line transplant) were selected in common among all experiments. GIHCG belongs to a family of non-coding RNA (ncRNA) and has been associated with prognosis in hepatocellular carcinoma [23] and colorectal cancer [24]. Other studies [25, 26] have revealed the role of GIHCG in key biological processes such as cell proliferation and cell migration in primary tumors and cancer cells.

Here, we observed GIHCG as differentially expressed (*p* < 0.05) among the non-sensitive and sensitive groups (see S1 Fig). Because GIHCG inhibits a cluster of miRNA which play important roles in regulating the expression of a number of genes, functional studies are required to expand the relative effects of GIHCG in multiple myeloma. It is worth mentioning that most patients in the sensitivity risk group received stem cell transplantation (see S2 Fig) as the first line in MM, which led to prolonged survival [27]. In addition, we recapitulate the prognostic value of serum beta 2 microglobulin in MM patients (see S3 Fig) in which the increased levels have been associated with a poor prognosis [28]. Taken together, these findings endorse our results and the predictive power of the MuLT-based model. The full list of gene and clinical markers is available in S3 File.

After CV, we observed that only a small number of genes appears in common among all independent CV rounds, highlighting the genetic heterogeneity in MM patients, which supports previous studies [12, 29, 30]. Of note, this combination of clinical markers and selected genes compose a completely new finding for predicting treatment sensitivity in MM.

**Fig 5:**
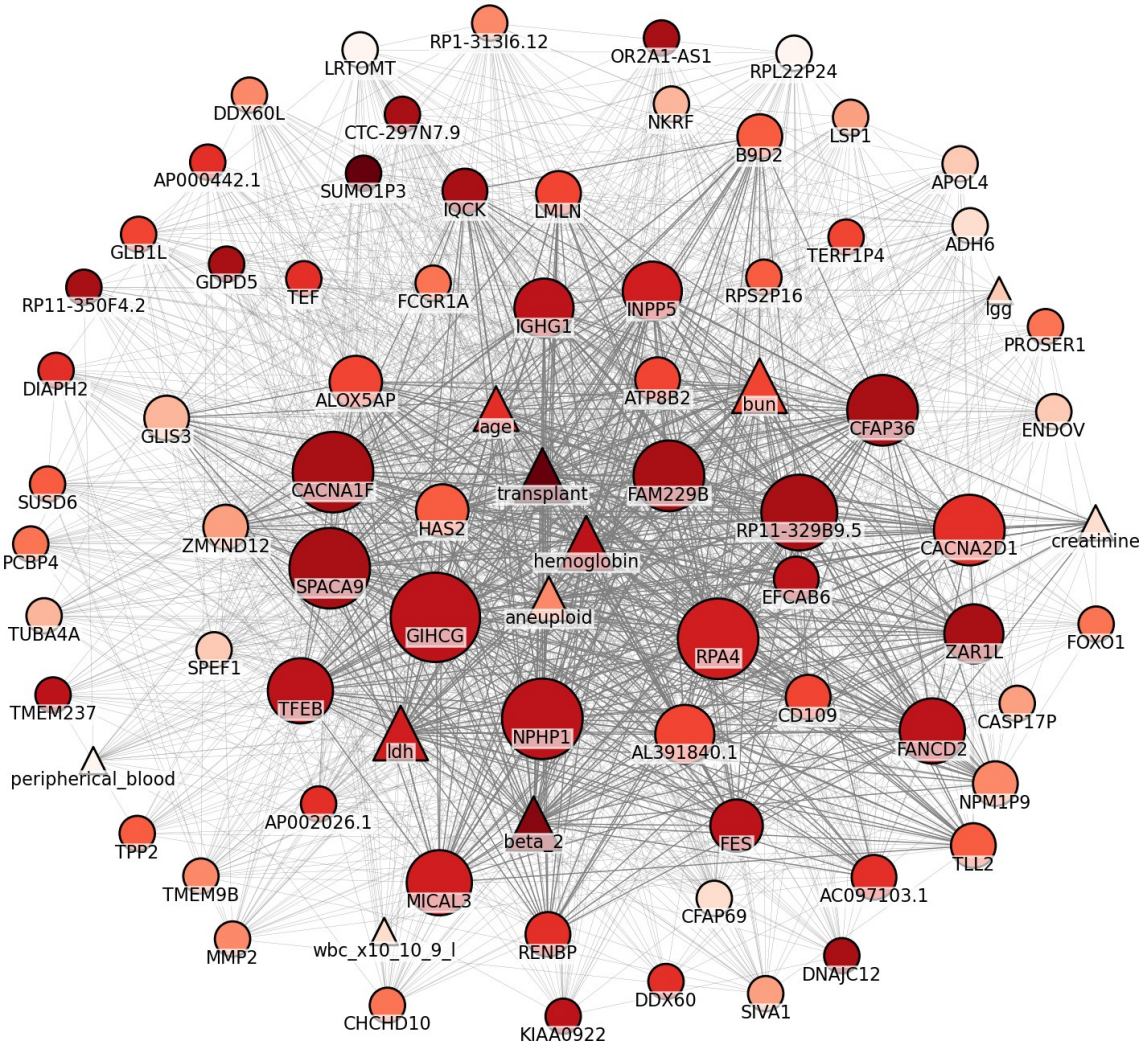
Relationship between clinical markers and genes represented as an undirected graph. Triangles and circles represent clinical markers and genes, respectively, while color intensity is inversely proportional to average p-value. The edge width corresponds to the number of CV rounds that the connected markers were selected together.

Fig 5 shows an undirected graph that describes the relationship among selected markers. To build the graph we used p-values computed in Algorithm 1, the number of independent CV rounds in which a marker was selected, and the marker category (i.e., clinical or gene). We built the graph based on the following rules:

1. Node size is proportional to the number of independent CV rounds that a marker was selected;
2. Clinical markers are represented as triangles while genes are represented as circles;
3. Node color intensity is inversely proportional to the average p-value of a marker among all independent CV rounds; and
4. Edge width is proportional to the number of CV rounds that two markers were selected together.

Remarkably, we reveal relationships among clinical markers and both known and unknown genes associated to MM, where the most relevant relationships for predicting TS are centralized (see S3 File for detailed graph structures).

### Simulations highlights the patients would have better sensitivity using a different treatment

**Fig 6:**
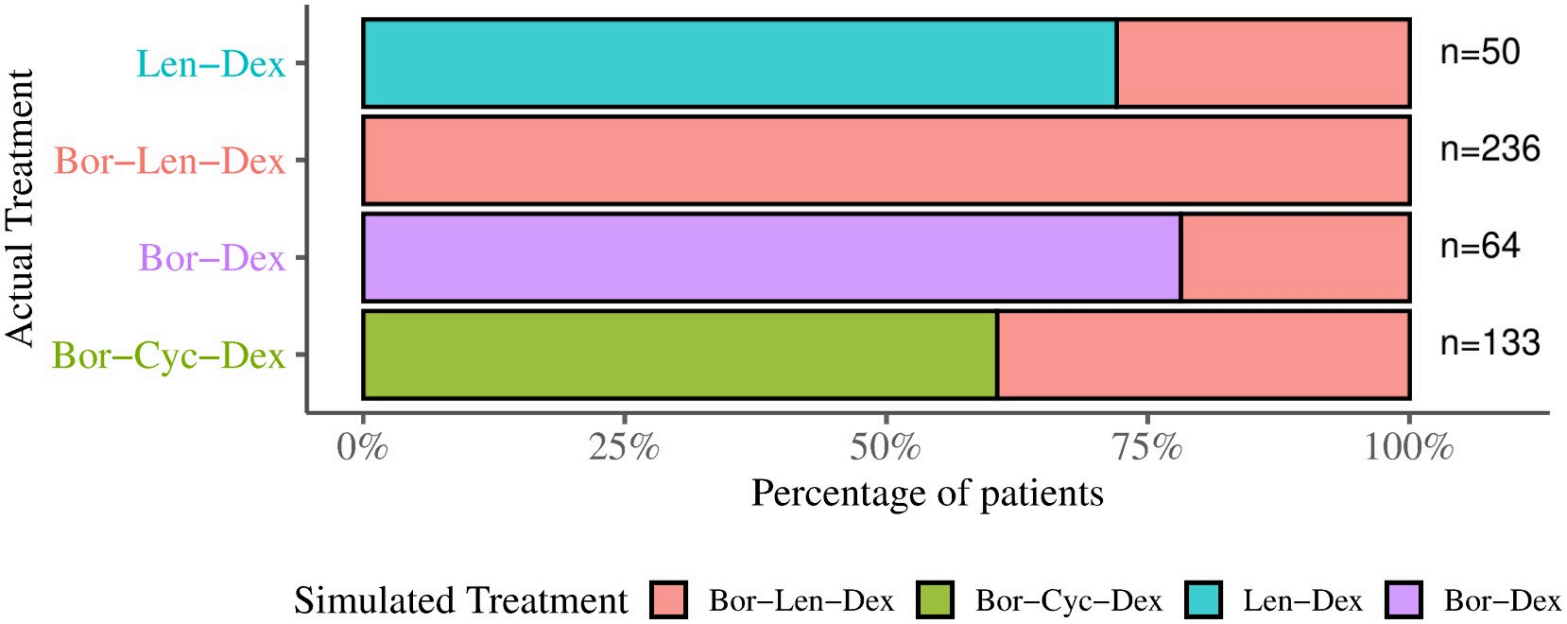
For each actual treatment (y-axis), we performed a simulation to identify the treatment (stacked bar colors) that maximizes the TS score considering the clinical and molecular data of MMRF cohort. The percentage of patients associated with a simulated treatment is shown on the x-axis.

Simulations were performed over the 10-fold CV experiments. For each experiment and patient in the validation data set, we employed the trained TS predictor to estimate the TS scores associated with different treatments (i.e., modifying the value of the input treatment marker and then predicting the TS score). Once this simulation is over, we have a data set of TS scores associated with patients and each possible treatment. For each patient, we chose simulated treatment as the one with the highest TS score. Finally, for each pair of treatments (*a*, *b*), we computed the percentage of patients that received treatment *a* while simulations indicated treatment *b* (i.e., treatment that maximize TS score). Fig 6 shows for each actual treatment (y-axis) the percent of patients (x-axis) associated with a simulated treatment (stacked bar color).

Simulation results indicate that 17.07% of patients would have better sensitivity using a different first line treatment (see Treatment Simulation section for details). Our approach sheds light on underlying aspects of disease heterogeneity, which allows further understanding of the interplay between clinical and molecular data coupled with treatment. See S4 File for detailed simulation results used to simulate the optimal treatment for each patient.

## Discussion

Identifying which treatment could maximize patient survival is a possible way to improve current clinical decision-making processes. In this study, we *(i)* investigated how well gene expression levels can predict FISH markers, *(ii)* estimated accuracy gain by adding genes to TS predictors based on clinical markers, *(iii)* defined MuLT to advance the state-of-the-art in MM TS prediction, and *(iv)* simulated MM optimal treatment in a personalized manner by using TS predictors. High quality data sets containing clinical markers, gene expression levels, and treatments related to the same patient and associated to TR clinical outcome are a requirement to create MM models that are helpful for understanding why patients that are expected to be sensitive to a certain treatment do not and vice-versa. Moreover, the data sets must have a large number of samples to allow ML algorithms to capture the complex interplay among the clinical and molecular data. We next restricted analysis to the MMRF data set, which contains data from more than one hundred different sites. To the best of our knowledge, there is no other public MM data set with the required quality and quantity.

Related work has performed analysis using different and independent data sets [12, 31, 32], either considering only genes or proposing independent models for each treatment. The MM Dream Challenge [33, 34] enables building around one hundred MM predictors based on a few different data sets, including MMRF. However, outcome measures are based on different survival thresholds, which bring an additional challenge with respect to predictor quality assessment. Additionally, AUC variations [33, 35] have been employed to deal with differences (e.g., survival thresholds) between outcome measures, which make comparisons more complex. Furthermore, different data sets of the MM Dream Challenge contain different marker sets, with some intersection between them, but generating several missing values when merging all data. The study herein does not try to address the described limitations in the related literature, but on establishing a preliminary understanding about the relation between clinical markers, gene expression levels, FISH, treatment, and sensitivity in a completely reproducible way. We pursue a systematic approach to create MM predictors.

Next steps are related to applying MuLT over different cancer data sets composed of clinical markers, gene expression levels, and treatment. This study was limited to binary classification, stratifying patients into either treatment sensitive or non-sensitive, but it could be generalized to perform regressions and multi-categorical classification. We are also interested in investigating more robust treatment representation. Techniques like Word Embedding [36] can be applied to create more robust representations for categorical markers (e.g., race, treatment), which can be helpful to reduce model noise and bias.

## Methods

### Data and processing

We extracted clinical markers, gene expression levels, FISH, treatments, and survival data from MMRF CoMMpass [37] (release IA14) data set composed of 1,525 patients. In order to ensure reproducibility of data collection and organization, we implement a tool called MMWebBot [38]. For our study, we only employed patients with clinical markers and gene expression levels associated with a non-missing value of the best response first line treatment. Patients associated with treatments with less than ten samples were excluded to avoid bias in CV experiments. Markers with more than 10% of missing values were also excluded. Based on these constraints, the analysed data set included 715 newly diagnosed valid MM samples, treated either with five different first line treatments, while non-treatment is assumed as valid first line treatment alternative (see Table 2 for detailed counts). Average age is 62.90, ranging from 27 to 93, 60.13% of the patients are male. The data set includes 55,103 genes, 26 clinical markers, and 17 FISH markers. Nominal markers (e.g., race) were represented as one-hot encoding. Each nominal value generated an individual binary marker, where 1 represents that the value is associated with the patient and 0 that it is not associated. Ordinal markers (e.g., stage) were represented as sequential integers starting from one. Missing values were replaced by zero.

To assess robustness and generalization, the data set was split into ten disjoint folds, stratified by treatments and TS outcome. Based on this setup, we performed ten individual experiments. For each experiment we used nine folds to perform model training, and one fold to perform inferences, simulations, and analyse results. All fold compositions are available at http://github.com/lucasvenez/mult.

### Treatment sensitivity outcome definition

In general, patients are associated with different clinical status (e.g., DDP, days to first response, days to overall survival, TR). This study was directed to modeling TS. For that, we created the TS outcome, that is defined from TR. We choose the mapping between TR and TS classes using Cox proportional hazards regression models (CHMs) [39]. Once TR outcome can be in one of six different classes, we consider five different TS outcome definitions. For each of these possibilities, we create a CHM, stratifying survival (DDP) by TS. We choose the TS definition that reached the minimum survival stratification p-value (see Fig 2).

### Simplified Machine Learning Approach (SMLA)

We defined a baseline pipeline called SMLA in order to perform three different analyses: (i) to identify if it is possible or not to predict structural genetics markers carried by FISH technology from gene expression levels—they are particularly relevant once these markers are broadly used in the current clinical decision-making process in MM; (ii) to measure the accuracy gain obtained by combining clinical markers and gene expressions levels; and (iii) to assess MuLT classification performance against a benchmark. Hence, SMLA is composed of three steps:

1. Marker selection based on our marker selection algorithm (see Algorithm 1);
2. Hyper-parameter optimization using the BO algorithm; and
3. Binary classifier (e.g., LightGBM, MLP, KNN) training.

For the first and second analyses we employed the LightGBM classifier, and for the third one we performed three independent experiments employing LightGBM, MLP and SVM classifiers. Table 3 presents the complete list of hyper-parameters optimized by the BO algorithm organized by method.

**Table 3:** List of optimized hyper-parameters per ML method.

Method	Hyper-parameter	Description
*LightGBM*	num_leaves	Max number of leaves in one tree
	scale_pos_weight	Weight of labels with positive class
	min_child_samples	Minimal number of data in one leaf
	bin_construct_sample_cnt	Number of data that sampled to construct feature discrete bins
	max_bin	Max number of bins that feature values will be bucketed in
	min_sum_hessian_in_leaf	Minimal sum hessian in one leaf
	bagging_fraction	Percentage of selected data without resampling
	feature_fraction	Percentage of features on each tree to be randonly selected
	feature_fraction_bynode	Percentage of features on each tree node to be randonly selected
*MLP*	hidden_layer_sizes	Number of neurons in the hidden layer
	learning_rate	Learning rate schedule for weight updates
	learning_rate_init	Initial learning rate
	max_iter	Maximum number of iterations
	tol	Tolerance for the optimization
*SVM*	C	Regularization parameter
	gamma	Kernel coefficient
	degree	Degree of the polynomial kernel function
	kernel	Kernel type to be used in the algorithm (i.e., linear, poly, rbf)

We employed a BO algorithm that uses Gaussian Processes (GP) [19] to minimize the log loss function *l* defined as:

$$\begin{array}{c} l\left(y,\hat{y}\right)=-\frac{1}{N}\sum_{i=1}^{N}y_{i}\times \text{log}\left({\hat{y}}_{i}\right)+\left(1-y_{i}\right)\times \text{log}\left(1-{\hat{y}}_{i}\right) \end{array}$$

where **y** represents expected values and $\hat{y}$ represents estimated values while *y*_*i*_ and ${\hat{y}}_{i}$ are the *i*^th^ elements of their corresponding vectors, and *N* is the size of vectors **y** and $\hat{y}$. The BO algorithm solves the following problem:

$$\begin{array}{c} \underset{H}{arg\;min}\;f\left(H;P,D\right) \end{array}$$

where *f*(⋅; ⋅, ⋅) returns the log loss after training a predictor *P*, given hyper-parameter values **H**, and a data set *D*.

For each learning method, BO initially generates 10 random **H** values that correspond to the hyper-parameters described in Table 3. It then computes *f*(**H**; *P*, *D*) and creates a surrogate function that fits the hyper-parameters values (**H**) to their resulting log loss. Based on the surrogate function, BO estimates new hyper-parameter values that are expected to return the global minimum log loss. It then computes *f* again for these estimated values and updates the surrogate function. This procedure is repeated over 50. Finally, BO returns the hyper-parameter values associated with the global minimum log loss. This process is used in all of our experiments that employ BO, the detailed implementation is available at https://git.io/JGBsv.

### Multi Learning Training (MuLT)

MuLT aims to create TS predictors that estimate if a patient is sensitive to a particular treatment based on clinical markers, gene expression levels, and treatment. As stated previously, our experiments were carried out using CV, splitting data into 10-folds. We used nine folds to compose the training data set and one to compose the validation data set. All steps described below were applied on training data, while the validation data was used only for inference and result analysis. Our novel ML-based approach starts by normalizing each value *m* of a marker *M* by *n*(*m*) = (*m*−*min*_*M*_)/(*max*_*M*_−*min*_*M*_). Both *max*_*M*_ and *min*_*M*_ are extracted from the training data set, and are also used to normalize the validation data set via *min*(1, *max*(0, *n*(*m*))). Minimum and maximum work for limiting output to the interval [0, 1].

Let **F** be a matrix composed of markers at columns and patients at rows, where each element *m*_*pM*_ describes the value of a marker *M* associated with a patient *p*. Let **c** be a vector associating a treatment sensitivity class (i.e., sensitivity and non-sensitivity) to a patient *p*. Based on normalized training data set, Algorithm 1 inputs **F** and **c**, and splits the patients along sets (rows of **F**) based on the clinical outcome class described by **c**. For each marker *M* (column of **F**), the algorithm tests whether these two classes originate from the same distribution based on KS test [40] for a significance level *α*, excluding markers with p-values higher than *α*. After that, the algorithm computes a pairwise linear correlation for remainder markers. For each marker $M_{i}^{\prime }$, the algorithm excludes $M_{i}^{\prime }$ if it has a linear correlation *β* > 0.75 with any marker $M_{j}^{\prime }\;\forall i\neq j$ with smaller p-value. The algorithm then outputs the selected markers **F**^**′**^. This marker selection algorithm is applied over genes (GS step) and clinical markers (CMS step) independently, generating selected genes **G**^**′**^, and selected clinical markers **C**^**′**^.

Our marker selection algorithm has two main objectives: (i) to identify which markers have different distributions when separated by TS classes; and (ii) to exclude markers that have their information encompassed by a more relevant marker. In order to implement the first objective, our algorithm applies a KS test between values of a marker among the TS classes. We also considered different approaches, testing the Maximal Information Coefficient (MIC) [41] with different thresholds to evaluate the effectiveness of a method that aims to identify non-linear correlation, and the Kruskal test [42] in order to evaluate the effectiveness of a non-parametric method. The second objective was addressed by using Pearson Correlation to measure linear correlation between markers to identify which of them are embedded in another that better discriminates TS classes. While designing MuLT, we did not identify relevant gains when using the different approaches above, but the reduced processing time when employing the KS test was notable.

**Algorithm 1** Pseudocode of the marker selection algorithm.

1: **function** MarkerSelection (**F**, **c**, *α*, *β*)

2:  *ExcludedMarkers* ← {}

3:  *AnalysedMarkers* ← {}

4:  *MarkerSet* ← *names*(**F**)       ⊳ Get marker names from matrix **F**

5:  **for all** *M*_1_ ∈ *MarkerSet* **do**        ⊳ For each marker *M*_1_, **do**

6:   *AnalysedMarkers* ← *AnalysedMarkers* ∪ {*M*_1_}    ⊳ Add *M*_1_ to *AnalysedMarkers*

7:   $p_{M_{1}}\leftarrow ComputeOrRetrievePvalue\left(F_{*M_{1}},c\right)$

8:   **if** *pM*_1_ > *α* **then**       ⊳ If p-value associated to marker *M*_1_ is greater than *α*

9:    *ExcludedGenes* ← *ExcludedGenes* ∪ {*M*_1_}   ⊳ Add *M*_1_ to *ExcludedMarkers* set

10:   **else**           ⊳ For each marker that was not analysed, do

11:    **for all** *M*_2_ ∈ *MarkerSet* \ *AnalysedMarkers* **do**

12:     $p_{M_{2}}\leftarrow ComputeOrRetrievePvalue\left(F_{*M_{2}},c\right)$

13:     **if** *pM*_2_ > *α* **then**

14:      *ExcludedMarkers* ← *ExcludedMarkers* ∪ {*M*_2_}

15:     **else**

16:      $pc\leftarrow |\;ComputePearsonCorrelation(F_{*M_{1}},F_{*M_{2}})\;|$

17:      **if** *pc* > *β* **then**       ⊳ If linear correlation is greater than *β*, then

18:       **if** *pM*_2_ ≥ *pM*_1_ **then**

19:        *ExcludedMarkers* ← *ExcludedMarkers* ∪ {*M*_2_}

20:       **else**

21:        *ExcludedMarkers* ← *ExcludedMarkers* ∪ {*M*_1_}

22:  *S* ← *MarkerSet* \ *ExcludedMarkers*

23:  **F′** ← F_*S_

24:  **return F′**

GP inputs the selected genes **G**′, applies it to estimate patient genetic profiles by using the k-means algorithm [43], and returns matrix $L=\left(l_{ij}\right)\in R^{m\times o}$ associating a patient *i* to its Euclidean distances from a cluster centroid *j*, where *m* is the number of patients.

Number of clusters *o* is defined by our Number of Clusters Selection (NCS) algorithm, which is based on the average Silhouette Coefficient (SC) [44]. NCS inputs selected genes **G**′ and iterates over the number of clusters *c* = 2, 3, … to compute a Silhouette Coefficient-based Metric (SCM), defined by

$$\begin{array}{c} SCM\left(c\right)=\frac{\bar{SC}\left(c\right)-\sigma_{SC}\left(c\right)}{\sigma_{NS}\left(c\right)+1} \end{array}$$

where $\bar{SC}\left(c\right)$ is the average SC of all observations for *c* clusters, *σ*_*SC*_(*c*) is the standard deviation of SC for *c* clusters, and *σ*_*NS*_(*c*) is the standard deviation of the number of samples in each cluster for *c* clusters. NCS stops after 10 iterations without getting a higher value for SCM, returning the number of clusters *o* = arg max_*c*_ *SCM*(*c*).

GC inputs the transpose of selected genes matrix **G**^′*T*^, estimates gene clusters using k-means algorithm, and returns a matrix $E=\left(e_{ij}\right)\in R^{m\times k}$ associating a patient *i* to the average expression level of each estimated gene cluster *j*, where *k* is the number of gene clusters defined by NCS algorithm.

GD inputs selected genes **G′** and returns a denoised representation **G**^*d*^ of selected genes. It is defined using a Deep Denoising Autoencoder (DDA) [45]. DDAs are composed of an input layer representing selected genes, five processing (hidden) layers, and an output layer representing the denoised selected genes. The number *i* of input and output units equals the number of selected genes. Processing layers has ⌊0.5*i*⌋, ⌊0.4*i*⌋, ⌊0.3*i*⌋, ⌊0.4*i*⌋, and ⌊0.5*i*⌋ units, where ⌊⋅⌋ is the floor operation. A DDA model is trained by adding a noise to the input and then reducing the Mean Squared Error (MSE) between the raw input and the DDA output. We modified the input values adding a noise generated from a random variable X∼N(0,1). We used the AdaDelta optimization algorithm and ReLU activation function [20] on processing units. Training was stopped after 1,000 iterations with no reduction of the minimum MSE loss. L2 regularization [20] was employed with a scale of 1%.

Finally, TSPT inputs the concatenation (**G**′ | **C**′ | **L** | **E** | **G**^**d**^ | **T**) and outputs a model able to predict an individual patient TS, where **T** is a matrix associating patients to its first line treatment. TSPT is defined by LightGBM [21] to model the individual patient TS. The training is composed of two parts. First, the training data is split into two folds and a hyper-parameter optimization (see LightGBM row in Table 3 for the complete list of optimized parameters) using the BO algorithm [19] is applied to define model parameter values to improve generalization and accuracy. One fold is used to train the model, and another to estimate the log loss. Hyper-parameter optimization returns the LightGBM parameters associated with the minimum average log loss in the 50 independent iterations. Taking the optimized parameters, the training data set is then split into three folds. An independent TS predictor is created based on each pairwise fold. Training is stopped after one iteration without log loss improvement, or after 100 iterations. Final TS score is defined via the average of TS scores computed by each predictor.

## Treatment simulation

We performed simulations based on MuLT predictors in order to identify which treatment could maximize the TS score for a given patient *p*, by computing:

$$\begin{array}{c} \kappa \left(G_{p},C_{p}\right)=\underset{t^{s}}{\text{arg}\;\text{max}}\;\text{MuLT}\left(G_{p},C_{p},t^{s}\right) \end{array}$$

where, for a given patient *p*, *t*^*s*^ is the simulated treatment, **G**_*p*_ is the gene vector, **C**_*p*_ is the clinical marker vector, and MuLT(⋅, ⋅, ⋅) is a trained predictor that returns a TS score.

Next, the relative number of patients that had a simulated treatment different from the actual one was computed by the equation below:

$$\begin{array}{c} \frac{1}{N_{p}}\sum_{p}\zeta \left(t_{a},\kappa \left(G_{p},C_{p}\right)\right) \end{array}$$

where *t*_*p*_ is the actual treatment for a given patient *p*, *N*_*p*_ is the number of patients in the cohort, *ζ*(*a*, *b*) is a function that returns 1 if *a* and *b* are not equal and 0 otherwise.

## Supporting information

S1 Fig Gene expression levels of GIHGC among non-sensitive and sensitive groups. (PDF)

S2 Fig Proportion of patients in the non-sensitive and sensitive groups who received stem cell transplantation per TS class. (PDF)

S3 Fig Levels of serum beta 2 microglobulin levels among non-sensitivity and sensitivity groups. (PDF)

S1 File Detailed performance metrics and lists of selected genes associated with each FISH predictor. (ZIP)

S2 File Detailed performance metrics and lists of clinical markers, and genes selected to compose TS predictors. (ZIP)

S3 File Graph structures of the selected clinical markers and genes. (ZIP)

S4 File Detailed results describing TS score for each treatment in a personalized manner. (ZIP)
