# Supplementary figures and images for: Machine learning predicts treatment sensitivity in multiple myeloma based on molecular and clinical information coupled with drug response
Source: PLoS One. 2021 Jul 28;16(7):e0254596. doi: 10.1371/journal.pone.0254596 (PMC8318243; doi:10.1371/journal.pone.0254596)

Wilcoxon,  $p = 0.042$

GIHCG

1000

500

0

Treatment Sensitivity

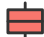

Non-sensitive

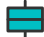

Sensitive

Supplement: S1 Fig — (PDF) [file pone.0254596.s001.pdf]

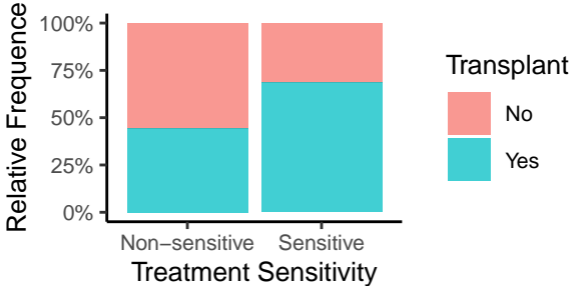

Supplement: S2 Fig — (PDF) [file pone.0254596.s002.pdf]

beta 2 microglobulin

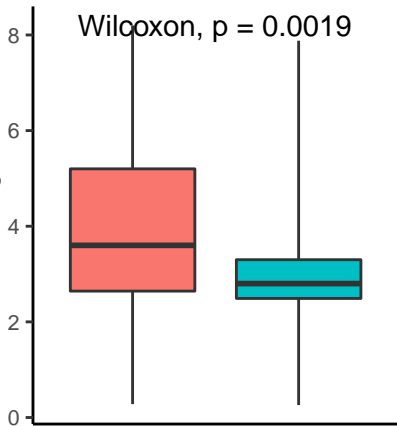

Treatment Sensitivity

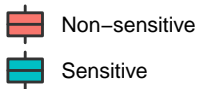

Supplement: S3 Fig — (PDF) [file pone.0254596.s003.pdf]
